# Editorial: The Role of Lipids in the Pathogenesis of Parkinson's Disease

**DOI:** 10.3389/fnins.2020.00250

**Published:** 2020-03-24

**Authors:** Veerle Baekelandt, Evy Lobbestael, Helena Xicoy, Gerard J. M. Martens

**Affiliations:** ^1^Laboratory for Neurobiology and Gene Therapy, Department of Neurosciences, Leuven Brain Institute, KU Leuven, Leuven, Belgium; ^2^Department of Cell Biology, Radboud Institute for Molecular Life Sciences, Radboud University Medical Centre, Nijmegen, Netherlands; ^3^Department of Molecular Animal Physiology, Faculty of Science, Donders Centre for Neuroscience, Donders Institute for Brain, Cognition and Behaviour, Radboud University, Nijmegen, Netherlands

**Keywords:** oxidative stress, lipids, neuroinflammation, Parkinson's disease, Parkinson's disease models, α-synuclein, ceramide, glucocerebrosidase

Parkinson's disease (PD) is the second most common neurodegenerative disease with a prevalence predicted to increase in the next decades. PD is characterized by both motor and non-motor symptoms that are linked to the progressive loss of dopaminergic neurons in the substantia nigra, the presence of Lewy bodies and neuroinflammation (Dexter and Jenner, 2013). Insight into disease etiology and pathogenesis has been obtained by genetic analyses, and studies using animal models of familial forms of PD, accounting for around 10% of the cases, and toxins that mimic some of the PD pathological hallmarks. Nevertheless, the exact molecular mechanisms underlying PD are elusive and disease-modifying treatments are not yet available. Interestingly, recent evidence indicates that lipids might play a crucial role in the pathogenesis of this devastating neurodegenerative disease (Klemann et al., 2017).

Lipids constitute a heterogeneous family of biomolecules mainly known by their role in energy storage, but they also represent crucial components of lipid membranes, lipid rafts, intracellular signaling pathways, and the immune system (Fernandis and Wenk, 2007; Welte and Gould, 2017). Mutations or single-nucleotide polymorphisms (SNPs) in genes encoding transcription factors and enzymes involved in lipid metabolism, such as *SREBF1* (Do et al., 2011)*, GBA* (Nichols et al., 2009), or *SMPD1* (Gan-Or et al., 2013), are associated with PD. Moreover, processes that are central in PD, such as the formation of α-synuclein aggregates, oxidative stress, and immune system activation, are modulated by lipids (De Pablo and Alvarez de Cienfuegos, 2000; Horvath and Daum, 2013; Galvagnion et al., 2016). Additionally, dietary intake of cholesterol and polyunsaturated fatty acids (PUFA), and application of cholesterol-lowering drugs (e.g., statins) seem to affect PD risk (Xicoy et al., 2019).

Despite the above, the role of lipids in PD has not been widely studied. Still, more insights into this domain may well lead to a better understanding of the disease, and the development of effective treatments and dietary interventions that may not only improve the quality of life of patients, but also slow down or even stop PD progression. We have therefore collected in this Research Topic a number of papers that cover various aspects of the role that lipids play in PD pathogenesis.

A general overview of the dysregulation of lipid metabolism and lipid pathways in PD is given by Alecu and Bennett. They provide a summary of the data obtained from animal models, and human brain and plasma samples, the genetic component of the association between PD and lipid metabolism and trafficking, and how key processes of PD pathology (i.e., oxidative stress and inflammation) can be triggered by lipid dysregulation. Moreover, the review emphasizes the physiological and pathological interactions between lipids and the classical PD-associated protein α-synuclein, the role of α-synuclein in lipid metabolism, and the interaction between the main genetic risk factor of PD (i.e., GBA, which encodes the enzyme glucocerebrosidase) and α-synuclein. In a similar way, Ikenaka et al. focus on the association between α-synuclein aggregation and cellular lipids. This review deals with the structural changes of α-synuclein induced by lipid membrane binding and glucosylceramide, which is the substrate of GBA. Additionally, the authors summarize the different forms of α-synuclein, and the role that the cellular environment and possibly lipids play in shaping these forms and as such define various synucleinopathies. In the third review, Plotegher et al. summarize the current knowledge on the association between the deregulation of ceramide metabolism and PD. The review presents an overview of the role of ceramides as structural and signaling molecules, and in cellular metabolism. Moreover, the authors discuss the genetic association between ceramides and PD, and how deregulated ceramide metabolism may alter brain homeostasis and lead to PD-associated neurodegeneration.

Canerina-Amaro et al. explore the changes in lipid raft microstructures during aging and following treatment with the neurotoxicant 1-methyl-4-phenyl-1,2,3,6-tetrahydropyridine (MPTP) in mice, and their correlation with α-synuclein aggregation. In the midbrain and cortex of aged and MPTP-treated mice, they observe alterations in the levels of gangliosides, cholesterol, PUFA and phospholipids. The variations in PUFA and phosphatidylserine correlate with α-synuclein aggregation and with a high abundance of α-synuclein phosphorylated at serine 129, a post-translational modification that promotes toxic aggregation. Interestingly, such changes are also present when comparing frontal cortex samples from PD patients with samples from controls. MPTP-treated mice are also used by Díaz et al. to investigate alterations in lipid composition of membrane microdomains, with a focus on two unaffected brain regions, namely the cerebellum and the occipital cortex. While they do not observe changes in the lipid profile of the cerebellum, they find alterations in the occipital cortex, including increased total mono-unsaturated fatty acids and total plasmalogens, a reduced PUFA level and a lower phospholipid-to-cholesterol ratio. The differences between the two brain regions are suggested to be due to the absence of the dopamine transporter in the cerebellum, which is necessary for MPTP neurotoxicity. Gaudioso et al. study the changes in lipid composition of the mitochondrial membrane in parkin null mice, which represent a genetic mouse model for PD. They observe an increase of phosphatidylethanolamine in young, but not old parkin null mice. On the other hand, old parkin null mice display increased hydroxylated ceramides levels, and a decrease of phosphatidylglycerol and phosphatidylinositol levels. These changes may affect both autophagy and mitophagy.

Laurence et al. wrote a hypothesis article that suggests a role in PD for the lipophilic fungus *Malassezia*, causative of seborrheic dermatitis. The authors argue that genetic susceptibility to PD is associated with increased lipid availability within cells as well as an abnormal immune response, which would allow the fungus to invade the central nervous system, and more specifically dopaminergic neurons. Dopaminergic neurons may be particularly susceptible to the invasion because L-DOPA, the precursor of dopamine, promotes *Malassezia* hypha formation and melanization, which could contribute to the pigmentation of the substantia nigra. Hence, the presence of *Malassezia* in the substantia nigra of PD patients deserves further attention.

The seven articles included in this Research Topic provide an overview of the current state of the art and present recent advances with respect to the role of lipids in PD pathology and in particular α-synuclein aggregation. With the lipid alterations observed in various models, including genetic and toxin-based mouse PD models as well as PD tissue samples, increasing evidence supports the hypothesis that lipids are important players in PD pathogenesis and additional future studies are thus warranted.

## Author Contributions

HX and GM made the first draft that was adjusted by VB and EL.

### Conflict of Interest

The authors declare that the research was conducted in the absence of any commercial or financial relationships that could be construed as a potential conflict of interest.
